# Training clinicians treating HIV to diagnose cytomegalovirus retinitis

**DOI:** 10.2471/BLT.14.142372

**Published:** 2014-09-22

**Authors:** David Heiden, NiNi Tun, Ernest Maningding, Matthew Heiden, Jennifer Rose-Nussbaumer, Khin Nyein Chan, Tamara Khizniak, Alexandra Yakubenko, Susan Lewallen, Jeremy D Keenan, Peter Saranchuk

**Affiliations:** a SEVA Foundation, 1786 Fifth Street Berkeley, CA 94710, United States of America (USA).; b Medical Action Myanmar, Yangon, Myanmar.; c Francis I Proctor Foundation, University of California, San Francisco, USA.; d Médecins Sans Frontières, Yangon, Myanmar.; e The Botkin Hospital for Infectious Diseases, St Petersburg, Russian Federation.; f Kilamanjaro Centre for Community Ophthalmology, Cape Town, South Africa.; g Southern Africa Medical Unit, Médecins Sans Frontières, Cape Town, South Africa.

## Abstract

**Problem:**

Acquired immunodeficiency syndrome (AIDS)-related cytomegalovirus (CMV) retinitis continues to be a neglected source of blindness in resource-poor settings. The main issue is lack of capacity to diagnose CMV retinitis in the clinical setting where patients receive care and all other opportunistic infections are diagnosed.

**Approach:**

We developed and implemented a four-day workshop to train clinicians working in human immunodeficiency virus (HIV) clinics how to perform binocular indirect ophthalmoscopy and diagnose CMV retinitis. Workshops comprised both classroom didactic instruction and direct clinical eye examinations in patients with advanced AIDS. Between 2007 and 2013, 14 workshops were conducted in China, Myanmar and the Russian Federation.

**Local setting:**

Workshops were held with local clinicians at HIV clinics supported by nongovernmental organizations, public-sector municipal hospitals and provincial infectious disease referral hospitals. Each setting had limited or no access to locally- trained ophthalmologists, and an HIV-infected population with advanced disease.

**Relevant changes:**

Clinicians learnt how to do binocular indirect ophthalmoscopy and to diagnose CMV retinitis. One year after the workshop, 32/38 trainees in Myanmar did systematic eye examination for early diagnosis of CMV retinitis as standard care for at-risk patients. In China and the Russian Federation, the success rates were lower, with 10/15 and 3/5 trainees, respectively, providing follow-up data.

**Lessons learnt:**

Skills necessary for screening and diagnosis of CMV retinitis can be taught in a four-day task-oriented training workshop. Successful implementation depends on institutional support, ongoing training and technical support. The next challenge is to scale up this approach in other countries.

## Résumé

**Problème:**

La rétinite à cytomégalovirus (CMV) liée au syndrome d'immunodéficience acquise (SIDA) continue à être une source négligée de cécité dans les zones à faibles ressources. Le principal problème est la capacité insuffisante à diagnostiquer la rétinite à CMV dans les établissements cliniques où les patients reçoivent des soins et où les autres infections opportunistes sont diagnostiquées.

**Approche:**

Nous avons développé et mis en œuvre un atelier de formation d'une durée de quatre jours pour former les cliniciens travaillant dans les cliniques traitant le virus de l'immunodéficience humaine (VIH) à effectuer une ophtalmoscopie indirecte binoculaire et à diagnostiquer la rétinite à CMV. Les ateliers comprenaient à la fois un enseignement didactique en salle de classe et des examens cliniques et directs des yeux chez les patients atteints du SIDA à un stade avancé. Entre 2007 et 2013, 14 ateliers de travail ont été organisés en Chine, au Myanmar et en Fédération de Russie.

**Environnement local:**

Les ateliers ont eu lieu avec des cliniciens locaux dans des cliniques traitant le VIH, soutenues par des organisations non gouvernementales, dans des hôpitaux municipaux publics et dans des hôpitaux provinciaux de référence pour les maladies infectieuses. Chaque établissement disposait d'un accès limité, voire d'aucun accès, à des ophtalmologistes localement formés et à une population infectée par le VIH à un stade avancé.

**Changements significatifs:**

Les cliniciens ont appris à effectuer une ophtalmoscopie indirecte binoculaire et à diagnostiquer la rétinite à CMV. Un an après l'atelier, 32/38 stagiaires du Myanmar effectuaient systématiquement l'examen des yeux pour le diagnostic précoce de la rétinite à CMV dans le cadre des soins standard pour les patients à risque. En Chine et en Fédération de Russie, les taux de succès ont été plus faibles, avec 10/15 et 3/5 stagiaires, respectivement, ayant fourni des données de suivi.

**Leçons tirées:**

Les compétences nécessaires pour le dépistage et le diagnostic de la rétinite à CMV peuvent être enseignées dans un atelier de formation spécialisée d'une durée de quatre jours. La réussite de la mise en œuvre dépend du soutien institutionnel, de la formation continue et du soutien technique. Le prochain défi est d'étendre cette approche dans d'autres pays.

## Resumen

**Situación:**

La retinitis por citomegalovirus (CMV) relacionada con el síndrome de inmunodeficiencia adquirida (SIDA) sigue siendo una fuente desatendida de ceguera en entornos con recursos insuficientes. El problema principal es la falta de capacidad para diagnosticar la retinitis por CMV en el entorno clínico donde los pacientes reciben atención y se diagnostican el resto de infecciones oportunistas.

**Enfoque:**

Desarrollamos y pusimos en práctica un taller de cuatro días para formar a médicos que trabajan en clínicas en las que se trata el virus de inmunodeficiencia humana (VIH) en la realización de la oftalmoscopia indirecta binocular y en el diagnóstico de la retinitis por CMV. Los talleres incluyeron una instrucción didáctica en el aula y exámenes oftalmológicos clínicos directos en pacientes con SIDA avanzado. Entre 2007 y 2013, se llevaron a cabo 14 talleres en China, Myanmar y la Federación de Rusia.

**Marco regional:**

Se realizaron talleres con médicos locales en las clínicas del VIH con el apoyo de organizaciones no gubernamentales, hospitales municipales públicos y hospitales provinciales de enfermedades infecciosas. Todos los emplazamientos presentaban un acceso a oftalmólogos formados a nivel local limitado o nulo y una población de personas infectadas por el VIH con enfermedad avanzada.

**Cambios importantes:**

Los médicos aprendieron a realizar la oftalmoscopia indirecta binocular y a diagnosticar la retinitis por CMV. Un año después del taller, 32/38 aprendices en Myanmar realizaban el examen oftalmológico sistemático para el diagnóstico precoz de la retinitis por CMV como parte de la atención estándar para los pacientes en riesgo. En China y la Federación de Rusia, las tasas de éxito fueron menores, con 10/15 y 3/5 aprendices, respectivamente, que proporcionaban datos de seguimiento.

**Lecciones aprendidas:**

Las competencias necesarias para la detección y el diagnóstico de la retinitis por CMV pueden enseñarse en un taller de formación especializada de cuatro días de duración. El éxito de la puesta en práctica depende del apoyo institucional, de la formación continua y de la asistencia técnica. El próximo desafío consistirá en extender este enfoque a otros países.

## ملخص

**المشكلة:**

ما زال التهاب شبكية العين الناجم عن الفيروسة المضخمة للخلايا ذات الصلة بمتلازمة العوز المناعي المكتسب (الأيدز) مصدراً مهملاً للعمى في البيئات فقيرة الموارد. وتتمثل المشكلة الرئيسية في الافتقار إلى القدرة على تشخيص التهاب شبكية العين الناجم عن الفيروسة المضخمة للخلايا في البيئة السريرية التي يتلقى فيها المرضى الرعاية ويتم تشخيص جميع حالات العدوى الانتهازية الأخرى.

**الأسلوب:**

قمنا بتنظيم وتنفيذ حلقة عمل لمدة أربعة أيام لتدريب الأخصائيين السريريين العاملين في عيادات فيروس العوز المناعي البشري على كيفية إجراء تنظير العين غير المباشر ثنائي العينية وتشخيص التهاب شبكية العين الناجم عن الفيروسة المضخمة للخلايا. واشتملت حلقات العمل على إرشادات تعليمية وفحوصات سريرية مباشرة لعيون مرضى الحالات المتقدمة من الأيدز. وبين 2007 و2013، تم إجراء 14 حلقة عمل في الصين وميانمار والاتحاد الروسي.

**المواقع المحلية:**

تم تنفيذ حلقات عمل مع تلقي الأخصائيين السريريين في عيادات فيروس العوز المناعي البشري المحليين دعماً من المنظمات غير الحكومية ومستشفيات البلديات التابعة للقطاع العام ومستشفيات إحالة الأمراض المعدية على مستوى المقاطعات. وكان الوصول إلى أخصائيي العيون المدربين على الصعيد المحلي محدوداً أو غير متوفر أصلاً في كل منطقة مع وجود سكان مصابين بحالات متقدمة من عدوى فيروس العوز المناعي البشري في كل منطقة.

**التغيّرات ذات الصلة:**

تعلم الأخصائيون السريريون كيفية إجراء تنظير العين غير المباشر ثنائي العينية وتشخيص التهاب شبكية العين الناجم عن الفيروسة المضخمة للخلايا. وبعد انقضاء عام واحد على حلقة العمل، أجرى 32 متدرباً من أصل 38 متدرباً في ميانمار فحصاً منهجياً للعين للتشخيص المبكر لالتهاب شبكية العين الناجم عن الفيروسة المضخمة للخلايا كرعاية قياسية للمرضى المعرضين للمخاطر. وفي الصين والاتحاد الروسي، انخفضت معدلات النجاح حيث قدم 10 متدربين من أصل 15 متدرباً و3 متدربين من أصل 5 متدربين، على التوالي بيانات المتابعة.

**الدروس المستفادة:**

يمكن تعلم المهارات اللازمة لتحري التهاب شبكية العين الناجم عن الفيروسة المضخمة للخلايا وتشخيصه في حلقة عمل تدريبية لمدة أربعة أيام يتم التدريب فيها على أداء مهام معينة. ويعتمد نجاح التنفيذ على الدعم المؤسسي والتدريب المستمر والدعم التقني. ويتمثل التحدي التالي في تسريع وتيرة هذا النهج في البلدان الأخرى.

## 摘要

**问题:**

在资源贫乏的环境中，艾滋病（AIDS）相关的巨细胞病毒（CMV）视网膜炎仍然是被疏忽的失明原因。主要问题在于缺乏在患者接受护理并诊断了所有其他机会性感染的临床环境中诊断CMV视网膜炎的能力。

**方法:**

我们开发和实施为期四天的讲习班来培训在艾滋病（HIV）诊所工作的临床医生如何执行双目间接检眼镜检查并诊断CMV视网膜炎。讲习班包括课堂理论教学和晚期艾滋病患者直接临床眼科检查。在2007年至2013年间，在中国、缅甸和俄罗斯联邦开展了14次讲习班。

**当地状况:**

讲习班在艾滋病诊所的当地医生中进行，得到非政府组织、公共部门市级医院和省传染病转诊医院的支持。每个诊所接触本地培训的眼科医师和晚期艾滋病感染人群的机会有限或没有这样的机会。

**相关变化:**

临床医师学会了如何进行双目间接眼底镜检查和诊断CMV视网膜炎。讲习班结束一年后，作为标准治疗，缅甸32名（共38名）学员对高危患者进行CMV视网膜炎早期诊断的系统视力检查。在中国和俄联邦的成功率较低，分别有10名（共15名）和3名（共5名）学员提供随访数据。

**经验教训:**

可以在为期四天的任务型培训讲习班教授CMV视网膜炎的筛查和诊断所必需的技能。成功实施取决于机构的支持、持续的培训和技术支持。面临的下一个挑战是在其他国家推广这种方法。

## Резюме

**Проблема:**

Цитомегаловирусный (ЦМВ) ретинит, ассоциированный с синдромом приобретенного иммунодефицита (СПИД), остается часто игнорируемой причиной слепоты в условиях ограниченности ресурсов. Основной проблемой является отсутствие возможностей по диагностике ЦМВ-ретинита в клинических условиях, в которых получают лечение пациенты и диагностируются все остальные оппортунистические инфекции.

**Подход:**

Был подготовлен и проведен четырехдневный семинар по обучению персонала клинических больниц по лечению ВИЧ методике бинокулярной обратной офтальмоскопии и диагностике ЦМВ-ретинита. Программа семинара предусматривала как инструктивные занятия в классах, так и непосредственно проведение проверки зрения пациентов с тяжелой формой СПИДа в клинических условиях. Всего на протяжении 2007-2013 гг. в Китае, Мьянме и Российской Федерации было проведено 14 семинаров.

**Местные условия:**

Семинары проводились с участием местных клинических врачей клинических больниц по лечению ВИЧ при поддержке неправительственных организаций, государственных муниципальных больниц и областных инфекционных лечебно-диагностических центров. В каждом случае у врачей не было возможности широкого доступа к услугам местных офтальмологов и больным ВИЧ с прогрессирующей стадией заболевания.

**Осуществленные перемены:**

Клинические врачи научились проводить бинокулярную обратную офтальмоскопию и диагностировать ЦМВ-ретинит. Через год после семинара 32 из 38 слушателей в Мьянме систематически проводили проверку зрения с целью ранней диагностики ЦМВ-ретинита в рамках стандартного обследования пациентов из группы риска. В Китае и Российской Федерации успехи были скромнее — данные для изучения предоставили 10 из 15 и 3 из 5 слушателей семинара соответственно.

**Выводы:**

Навыки, необходимые для обследования и диагностики ЦМВ-ретинита, могут быть получены в ходе четырехдневного целевого учебного семинара. Успех внедрения зависит от институциональной и технической поддержки, а также непрерывного обучения. Следующей задачей является расширение этого подхода с охватом других стран.

## Introduction

Acquired immunodeficiency syndrome (AIDS)-related cytomegalovirus (CMV) retinitis is a potentially blinding opportunistic infection that used to occur in up to one-third of HIV-infected patients in high-income countries before the availability of antiretroviral therapy (ART). It accounts for over 90% of the blindness related to human immunodeficiency virus (HIV) infection.^1^ CMV retinitis has virtually disappeared in high-income countries due to the routine early diagnosis of HIV infection and initiation of ART. Now, CMV retinitis primarily affects HIV-infected patients in middle- and low-income countries who are diagnosed with advanced immunodeficiency (late presenters) in settings with limited resources or poor access to care.^2^^,^^3^

The fundamentals of CMV retinitis management are early diagnosis, specific anti-CMV treatment and ART.^4^ Diagnosis is achieved by clinical examination; the gold standard is binocular indirect ophthalmoscopy through a dilated pupil, performed by a trained examiner.^3^ Early diagnosis requires systematic screening of all patients with CD4+ T lymphocyte counts less than 100 cells/μL, because retinal damage may already be irreversible and extensive by the time the patient first reports symptoms.^5^ Unfortunately, ophthalmological care is generally not available to HIV patients in resource-poor settings because of stigma associated with the disease and the logistical challenges of referring patients who are gravely ill. Ophthalmologists are often not available at all, or are not motivated or trained to treat HIV-related eye complications. Since ophthalmologists are traditionally the only clinicians trained in both indirect ophthalmoscopy and diagnosis of CMV retinitis, timely ophthalmic consultation for diagnosis of CMV retinitis in resource-poor settings is virtually never achieved.^6^

In a wide selection of settings, we have directly observed the lack of diagnostic capacity, absence of systematic screening of high-risk patients, and dire clinical consequences of delayed diagnosis of CMV retinitis.^3^ A growing body of evidence demonstrates poor clinical outcomes in CMV retinitis, with 21–36% of eyes already blind when the patient is first examined by an ophthalmologist.^3^^,^^7^ An increasing number of patients are successfully being treated for HIV, yet left permanently blind,^8^ and there has been no apparent decrease in the burden of CMV retinitis over the past decade.^2^

## Approach

During a 2006 evaluation of AIDS-related eye complications conducted by an ophthalmologist in HIV clinics in Myanmar, the high prevalence and consequences of failure to diagnose CMV retinitis became apparent. Therefore, a short, goal-oriented workshop in collaboration with other clinicians and ophthalmologists was devised for training non-ophthalmologist HIV clinicians to perform indirect ophthalmoscopy and diagnose CMV retinitis. This workshop was supported by the SEVA Foundation, Médecins Sans Frontières, Medical Action Myanmar and other nongovernmental organizations (NGOs). The workshop model evolved and improved over the course of 14 iterations in China, Myanmar and the Russian Federation; the current version is described in Box 1.

Box 1 **Workshop description**

Between three and six HIV/AIDS clinicians are enrolled for each workshop. Trainees are selected based on interest in learning eye examination skills, high motivation, at least one year of HIV clinical experience, an ongoing position in a clinical HIV programme where their skills will be needed and practised, and ideally younger than 30 years. The lead ophthalmologist and trainer is trained in uveitis and experienced in AIDS-related eye disease; one or two other ophthalmologists commonly provide teaching support. During the workshop each trainee has exclusive use of a portable battery-powered indirect ophthalmoscope (ScanOptics, Adelaide, model SO-2200), a 28D lens, and a homemade model eye.^9^ One month before the workshop, the series of lectures comprising the workshop curriculum is emailed to each trainee for independent study.

The first morning comprises six short lectures on ocular anatomy, basic bedside examination of the eye and binocular indirect ophthalmoscopy, followed by practice with model eyes. The second and third mornings comprise a series of lectures on CMV retinitis and the differential diagnosis of AIDS-related retinal disease. The initial six lectures are presented to the group by the trainees. Mornings begin with discussion of problems and written tests on prior material. The final morning begins with a written examination based on retinal photographs, emphasizes common patient management scenarios, and is followed by lengthy discussion. Throughout the workshop, whenever time is available, the trainers lead teaching sessions based on photographic examples of pathology. Each afternoon (3–5 hours) trainees examine dilated eyes of patients either known to have CMV retinitis or at high-risk of AIDS-related retinal disease. A typical workshop has five patients the first day and 10 on each of the following three days. Trainees examine and make retinal drawings of both eyes of every patient. During the first 1–2 days, trainees bring their model eye with them to the bedside and go back and forth from model eye to patient whenever they encounter technical difficulty. Trainees review lecture material each evening and are required to spend at least 30 minutes practising indirect ophthalmoscopy with model eyes during the first two evenings. There are final written course evaluations by each trainee, and by the lead trainer, for consideration in improving the next workshop.

AIDS: acquired immunodeficiency syndrome; CMV: cytomegalovirus; HIV: human immunodeficiency virus.

Workshop success is based on three key factors. First, the technique of indirect ophthalmoscopy is divided into small, well-described steps, accompanied by intensive practice with model eyes, and supported by four days of individual attention from trainers. Second, the didactic material is highly repetitive and the curriculum has a narrow focus on material necessary for diagnosis of serious AIDS-related opportunistic infections of the eye: CMV retinitis, HIV retinopathy, choroidal tuberculosis, syphilis, necrotizing herpetic infection, toxoplasmosis and myelinated nerve fibre layer. This narrow focus is logical and appropriate, given the extremely high pre-test probability that any white lesion diagnosed by indirect ophthalmoscopy in a patient with advanced AIDS will be either CMV retinitis or a cotton-wool spot.^10^ The curriculum is not designed for training clinicians in primary eye care. Third, the workshop is based on active individual and group participation, and immediate immersion in clinical care. Trainees, both novice and experienced, participate in the didactic programme by presenting most lectures, and they spend over half their time examining and diagnosing patients. Experiential learning begins with clinical examinations on the first day and provides immediate context for future didactic material.

## Relevant changes

To evaluate the implementation of CMV retinitis screening programmes following the workshops, we contacted former trainees and their supervisors to review self-reported clinical performance in the first year after training. We trained 65 people over the 14 workshops, of these, 58 were expected to provide clinical screening for CMV retinitis (two trainees failed to demonstrate satisfactory qualifications, and five were HIV advisors or administrators taking the course for educational purposes). We received information either from the trainee or a supervisor for 52 of the 58 (89.6%) successful trainees. Of these 52 trainees, 45 (86.5%) performed CMV retinitis screening in the first year after the training. During this year, a median of 120 screening examinations (interquartile range, IQR: 61–300) were performed and a median of 15 (IQR: 6–40) cases of CMV retinitis were diagnosed per trainee (Table 1).

**Table 1:** Clinical impact one year after completion of a four-day training workshop for eye examination and diagnosis of cytomegalovirus retinitis, China, Myanmar and the Russian Federation, 2007–2013

Metric	China (2009, 2010, 2013)	Myanmar (2007–2013)	Russian Federation (2011, 2012)
No. of workshops	3	9	2
No of trainees			
Enrolled in workshop	16	44	5
Satisfactorily completed workshop	15	43	5
Were expected to perform screening^a^	15	38	5
Have follow-up data available	11	38	3
Performed screening during first year	10	32	3
HIV patients screened, median (IQR)^b^	73.5 (55–100)	230.5 (100–430)	40 (30–40)
Patients diagnosed with CMV retinitis, median (IQR)	6.5 (5–20)	20 (9.5–47.5)	4 (0–4)

CMV: cytomegalovirus; HIV: human immunodeficiency virus; IQR: interquartile range.

^a^ Advisors and administrators occasionally completed the workshop; these individuals were not expected to perform screening.

^b^ Of those who performed screening, self-reported by each trainee or a supervisor.

Workshops have been conducted in Myanmar since 2007. This has directly led to systematic screening for CMV retinitis in HIV clinics for the majority of patients at risk enrolled in HIV treatment nationally. Screening coverage extends over most of the country, including areas in conflict. At the end of 2013, 67 643 patients were under treatment with ART in Myanmar, with 37 500 (55%) of these patients enrolled in the NGO programmes that provide routine screening for CMV retinitis. This success has occurred within the institutional structure of well-supported NGO programmes, with highly motivated clinicians, and with ongoing training and technical support.

In China, despite difficult communication and the almost complete lack of opportunity to provide technical support after the workshop, there was apparent evidence of success. Most trainees (10/11) provided information that they have implemented eye examination in their clinical care and are diagnosing cases of CMV retinitis. We had limited opportunity to guide trainee selection before the workshops, and several trainees were either unsuited to implementing retinal screening in their setting, did not work regularly in an HIV clinic or subsequently left the country to study overseas.

There was initial success in the Russian Federation. However, it was not sustained, due to trained clinicians being transferred, political difficulties in providing technical support, ambiguous health-care policies regarding eye skills for HIV clinicians and weak institutional support.

## Discussion

Although this initiative is not part of current World Health Organization policy,^11^ we believe that examination of the retina by indirect ophthalmoscopy should be part of the standard initial physical examination for all HIV-infected patients who first present with advanced immunodeficiency. About 35% of individuals infected with HIV in low-income countries have a CD4+ T lymphocyte count less than 100 cells/μL before starting ART treatment; these are patients at risk for CMV retinitis.^12^ Our experience, gathered over more than a decade, supports the strategy of clinicians diagnosing and treating CMV retinitis at the primary care level, as they currently diagnose and treat all other major opportunistic infections. We found that HIV clinicians in all three settings were generally motivated and enthusiastic to learn eye examination skills, and could be trained to accurately diagnose CMV retinitis. Furthermore, we found that clinicians completing the workshop had good agreement with expert ophthalmologists regarding the diagnosis of CMV retinitis.^13^ This is consistent with a recent systematic review that found no difference in the reported prevalence of CMV retinitis, whether screening was performed by an ophthalmologist or by an HIV clinician trained in retinal examination.^2^

Once diagnosis of CMV retinitis is achieved, timely and appropriate anti-CMV treatment can be provided. The issue of treatment is not covered in this report, except to note that both intravitreal injection of ganciclovir and oral treatment with valganciclovir can be provided by trained clinicians at the primary care level.^13^^,^^14^

Routine point-of-care eye examination for HIV late presenters provides ancillary benefits. There is evidence that indirect ophthalmoscopy can immediately identify some patients with disseminated tuberculosis if choroidal tubercles are seen, allowing for earlier diagnosis and treatment.^15^ Finally, cotton-wool spots, strongly associated with high HIV viral load,^16^ may be clinically helpful for monitoring response to ART in settings without access to viral load testing, identifying patients who are non-adherent or who have a drug-resistant strain of HIV.

In conclusion, one of the main lessons learnt (Box 2) is that a well-designed four-day workshop is feasible and effective for training clinicians to perform indirect ophthalmoscopy and diagnose CMV retinitis. Clinical impact depends on the institutional support provided by the health-care system, and sustained training and technical support. To scale up these workshops, we need to provide widely accessible didactic materials; develop methods to systematically monitor clinical performance, and offer mentorship in the months following the workshop (e.g. via telemedicine); and identify clinicians who are willing and able to be future trainers. If these challenges can be met, we believe the fundamental necessary innovation of transferring ophthalmic skills and primary management of CMV retinitis to the HIV clinician will reduce the prevalence of AIDS-related blindness in middle- and low-income countries.

Box 2 **Summary of main lessons learnt**

- A four-day training workshop can teach the skills of indirect ophthalmoscopy and diagnosis of CMV retinitis to motivated clinicians who can then successfully screen patients for AIDS-related CMV retinitis at the primary care level.
- One year after the workshop most of the trainees were providing systematic eye examination for early diagnosis of CMV retinitis as standard care for at-risk patients.
- Successful implementation depends on institutional support, ongoing training and technical support.
